# Linking the oceans to public health: current efforts and future directions

**DOI:** 10.1186/1476-069X-7-S2-S6

**Published:** 2008-11-07

**Authors:** Hauke L Kite-Powell, Lora E Fleming, Lorraine C Backer, Elaine M Faustman, Porter Hoagland, Ami Tsuchiya, Lisa R Younglove, Bruce A Wilcox, Rebecca J Gast

**Affiliations:** 1 Marine Policy Center, Woods Hole Oceanographic Institution, Woods Hole, Massachusetts, USA; 2 Departments of Epidemiology & Public Health and Marine Biology & Fisheries, Miller School of Medicine and Rosenstiel School of Marine and Atmospheric Sciences, University of Miami, Clinical Research Building, 10th Floor (R669), 1120 NW 14th Street, Miami, Florida, USA; 3 National Center for Environmental Health, US Centers for Disease Control and Prevention, 4770 Buford Highway NE, MS F-57, Chamblee, Georgia, USA; 4 Center on Human Development and Disability, University of Washington, Seattle, Washington, USA; 5 Pacific Northwest Center for Human Health and Ocean Studies, Institute for Risk Analysis and Risk Communication, University of Washington, Seattle, Washington, USA; 6 Department of Tropical Medicine, Medical Microbiology and Pharmacology, John A. Burns School of Medicine, University of Hawaii, Honolulu, Hawaii, USA; 7 Biology Department, Woods Hole Oceanographic Institution, Woods Hole, Massachusetts, USA

## Abstract

We review the major linkages between the oceans and public health, focusing on exposures and potential health effects due to anthropogenic and natural factors including: harmful algal blooms, microbes, and chemical pollutants in the oceans; consumption of seafood; and flooding events. We summarize briefly the current state of knowledge about public health effects and their economic consequences; and we discuss priorities for future research.

We find that:

• There are numerous connections between the oceans, human activities, and human health that result in both positive and negative exposures and health effects (risks and benefits); and the study of these connections comprises a new interdisciplinary area, "oceans and human health."

• The state of present knowledge about the linkages between oceans and public health varies. Some risks, such as the acute health effects caused by toxins associated with shellfish poisoning and red tide, are relatively well understood. Other risks, such as those posed by chronic exposure to many anthropogenic chemicals, pathogens, and naturally occurring toxins in coastal waters, are less well quantified. Even where there is a good understanding of the mechanism for health effects, good epidemiological data are often lacking. Solid data on economic and social consequences of these linkages are also lacking in most cases.

• The design of management measures to address these risks must take into account the complexities of human response to warnings and other guidance, and the economic tradeoffs among different risks and benefits. Future research in oceans and human health to address public health risks associated with marine pathogens and toxins, and with marine dimensions of global change, should include epidemiological, behavioral, and economic components to ensure that resulting management measures incorporate effective economic and risk/benefit tradeoffs.

## Background

The oceans are connected to public health on several levels. The health and wellbeing of individuals and populations can be affected by ocean conditions, resources, and phenomena in both positive and negative ways. This paper reviews some of the significant linkages between oceans and public health, highlights recent research on this topic, and suggests possible priorities for future work in this area.

**Figure 1:**
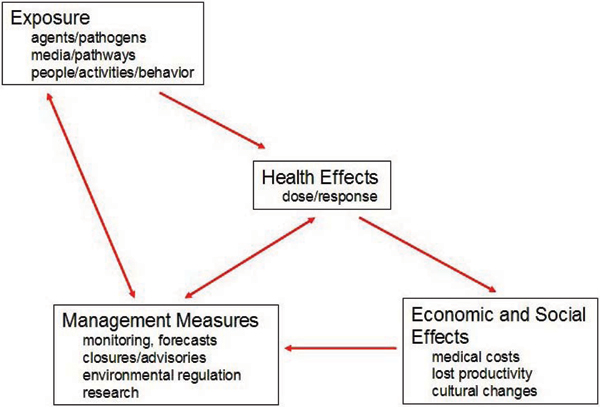
Framework for ocean-related health effects, consequences, and management.

Figure 1 illustrates one way of thinking about ocean-related health effects, their consequences, and the efforts to manage them. People are exposed to environmental conditions, nutrients, pathogens, and other agents associated with the oceans through a variety of media and pathways, including direct contact with or ingestion of sea water during work, recreation, or inundation events; consumption of seafood; and exposure to ocean-borne agents in near-shore sand, sediments, or air. Depending on the nature of the exposure and the characteristics of the exposed populations, this exposure leads to health effects that may be negative (e.g., gastro-intestinal illness, toxic poisoning, drowning) or positive (e.g., nutritional benefits of seafood, health benefits from marine recreation or from marine-derived pharmaceuticals). These health effects in turn have economic consequences (e.g., cost of medical care, lost productivity, medical costs avoided through exercise), as well as other social effects (e.g., changes in cultural traditions or trades related to marine resources or the marine environment). The health consequences and their social and economic ramifications lead society to adopt management measures to address exposure and health effects. For example, several coastal states operate monitoring programs for harmful algal bloom organisms (HABs) and their toxins to prevent the harvesting and consumption of contaminated shellfish, and associated shellfish poisoning in humans.

In the following sections, we provide brief reviews of the state of knowledge about human/public health outcomes associated with HABs, microbes, and chemical pollutants in the oceans; with flooding and inundation events; and with the consumption of seafood; and we review the implications for management measures and future research priorities. While this is not an exhaustive list of all linkages between the oceans and public health, it captures the major connections. We also discuss future research priorities in oceans and human health as a new scientific discipline.

Although we do not treat them in great detail in this paper, the marine dimensions of global climate change, such as ocean warming, sea level rise, and changes in ocean chemistry driven in part by increases in atmospheric greenhouse gas concentrations, can influence all of these linkages (as well as other aspects of marine ecosystems less directly linked to human health). For example, changing marine conditions can shift traditional ranges of marine species and promote or compromise their prevalence, and can alter coastal environments by changing weather patterns (including severe weather events) and shifting the coastline. Some populations, such as islanders and coastal groups that depend heavily on local marine resources, may be particularly vulnerable to health effects from this kind of change. We conclude that global change should be seen as a potentially pervasive factor influencing the future nature of all connections between oceans and human health.

## Adverse health outcomes from HABs, microbes, and chemical pollutants

Much of the public health research conducted to date on linkages between the oceans and human wellbeing has focused on the potential adverse health risks for humans and other animals associated with exposures to harmful algal blooms and their toxins, to microbes (i.e., bacteria, viruses, and parasites), and to anthropogenic chemicals [1-5].

### Harmful algal blooms

Harmful algal blooms (HABs), some of which are also known as "red tides," are exuberant growths of phytoplankton (such as diatoms, dinoflagellates, and cyanobacteria) in aquatic environments. These blooms are considered "harmful" when they create public health risks or adversely affect the local ecology by producing very potent natural toxins, depleting oxygen, or blocking the sunlight from reaching the lower depths of the water column. The primary adverse impacts on humans and other animals occur through exposure to the natural HAB-generated toxins which are potent neurotoxins, hepatotoxins, dermatotoxins, and in some cases, carcinogens (see reviews by Fleming *et al. *[6], Backer *et al. *[7,8], and Judd *et al. *[9]).

People and other animals (including fish, birds, and marine mammals) are exposed to and harmed by HAB toxins. Exposures occur when people or other animals eat contaminated food, drink contaminated water, contact contaminated water with their skin, or inhale contaminated aerosols. These toxins can cause acute and chronic effects, and high exposures can be lethal. As investigators improve the characterization of specific HAB organisms, they have found that many species are capable of producing more than one toxin. In addition, new congeners of well-known toxins are being identified, making the association between a specific exposure and a specific health outcome in some cases difficult to assess [6-8].

New methods are being developed to determine when a HAB event has occurred. Biosensors that allow real-time monitoring can be used to detect HAB toxins and help managers take actions such as posting warnings on beaches or closing shellfish beds to prevent human exposure to these toxins [10,11].

### Chemical agents and waterborne pathogens

In addition to HABs, there are other threats associated with exposure to coastal waters. These threats include pathogens (such as bacteria, viruses, and parasites) associated with fecal contamination of water; and anthropogenic chemicals (persistent organic pollutants [POPs], pharmaceutically active products, and heavy metals such as mercury) associated with industrial waste effluents. The primary sources of these chemical and microbial ocean-borne threats are anthropogenic activities that generate both point and non-point pollution, such as combined sewer overflows, wastewater treatment failures, permitted and non-permitted industrial discharges, and coal-burning power generation. There are also naturally-occurring water-borne chemical toxicants and pathogens that can adversely affect people who use the water; these include arsenic (a heavy metal) and vibrios (bacterial pathogens). Seasonal cholera outbreaks in South Asia are associated with plankton blooms that include *V. cholerae*'s natural host reservoir, copepods [12]. Many other pathogens of zoonotic origin have become an increasing concern, though marine mammals currently appear to be at greatest risk [2]. The numerous pathogens associated mainly with feral, agricultural, and domestic animals include a wide range of waterborne parasitic and bacterial pathogens and enteric viruses [13-15].

While some water contaminants may be toxic, others are potentially useful compounds, such as marine-derived pharmaceuticals. For example, Abraham *et al. *[16] recently reported finding a naturally occurring, potent antagonist to the brevetoxins associated with Florida red tides that may be useful in treating cystic fibrosis.

### Susceptible populations and media/pathways

The scale of the population that potentially may be exposed to toxins from HABs, microbes, and chemical pollutants in the oceans is large. For example, 62 million Americans are estimated to swim in the nation's coastal waters; and Americans spend more than 800 million person-days at the beach annually [17].

Assessing the human health risk associated with exposure to water-borne pathogens and toxins requires information on the susceptibility of individuals in the target population. Healthy people may respond to exposure to an environmental contaminant with self-limiting symptoms that do not interfere with day-to-day activities. However, people with underlying diseases or inherent genetic susceptibilities may react differently to equivalent exposures. The response to a given environmental exposure may depend on an individual's genetic makeup, physiological characteristics, and personal lifestyle as well as the route of exposure, the dose, and the specific physiologic outcome.

In general, young children are considered a susceptible population because their size or behavior may result in a relatively greater dose for a given exposure [18]. For example, small children are more likely than older children or adults to engage in hand-to-mouth behavior that puts them at increased risk of swallowing pathogen-contaminated recreational beach waters. Fetuses are also more susceptible because their physiologic systems are developing rapidly and can be exquisitely sensitive to disruptions induced by environmental contaminants. In addition, *in utero *exposures may put an individual at increased risk from future toxic insults, such as exposure to carcinogens [18].

Another group of people particularly susceptible to environmental toxicants and contaminants comprises those with depressed immune function (e.g., people with HIV/AIDS and those in chemotherapy treatment). Specific recommendations warn these groups to avoid contact with pathogen-contaminated waters for drinking and recreational activities. Other populations generally considered to be susceptible to adverse health outcomes from a variety of exposures include pregnant women (in large part due to the risk to the unborn fetus), the elderly, and persons with underlying chronic diseases.

In addition to people whose personal physiologic or genetic characteristics increase their risks from environmental exposures, a separate category of susceptible populations includes people who are dependent on a particular resource for socio-economic or other cultural reasons. People who cannot understand health warnings due to language and cultural barriers or whose livelihood is closely linked with a specific traditional environment are particularly at risk for adverse effects of local environmental contamination. For example, Native American groups in the Pacific Northwest have been concerned about domoic acid contamination of razor clams, which are a traditional subsistence food and economic resource for the communities [6,19-22].

Indications of the adverse effects from environmental contamination can also be observed in animals that depend on a specific environment to survive. Adverse human health effects associated with changing environmental risks may be predicted by what is observed in sentinel animal species. For example, both individually and in combination, the HAB toxins (i.e., domoic acid and brevetoxin), anthropogenic chemicals, and even human-associated pathogens have been shown to severely affect the health of the California sea lion and other marine mammals [23-25].

### Health effects and routes of exposure

Comprehensive lists of HAB organisms, the known toxins they produce, and the known diseases resulting from exposure have been published elsewhere [6-8]. Eating seafood contaminated with neurotoxins elaborated by dinoflagellates and diatoms is associated with the most well described of these diseases, including paralytic shellfish poisoning (PSP), neurotoxic shellfish poisoning (NSP), diarrheic shellfish poisoning (DSP), amnesiac shellfish poisoning (ASP), and ciguatera fish poisoning (CTX). Cyanobacterial (blue green algal) toxins have been associated with gastrointestinal, neurotoxic, and hepatotoxic effects in animals and humans after skin contact with or consumption of contaminated water (see Table 1). Laboratory studies have shown that toxins elaborated by cyanobacteria are genotoxic and tumor-promoting and can induce kidney damage. Chronic neurologic diseases (such as amyelotrophic lateral sclerosis [ALS], Parkinson's Disease, and Alzheimer's dementia) may be associated with the consumption of a neurotoxic cyanobacterial toxin, beta-N-methylamino-L-alanine (BMAA), in humans and possibly other animals [26,27]. In addition to exposure through eating or drinking contaminated food and water, investigators have recently described increased respiratory symptoms and pulmonary effects from exposure to aerosolized brevetoxins associated with Florida red tides from the dinoflagellate *Karenia brevis *(see HAB Case Study).

**Table 1:** Summary of harmful algal bloom (HAB) organisms that pose health threats to humans, the toxins they produce, and the diseases associated with exposure to the toxins.

**Representative organism**	**Toxins elaborated**	**Disease**
Diatoms: *Pseudo-nitzschia *spp.	Domoic acid	Amnesiac shellfish poisoning
Dinoflagellate: *Karenia brevis *(formerly *Gymnodinium breve*)	Brevetoxins	• Neurotoxic shellfish poisoning • Florida Red Tide Respiratory Irritation
Dinoflagellates: *Gymnodinium catenatum, Pyrodinium bahamense *var. *compressum, Alexandrium *spp.	Saxitoxins	Paralytic shellfish poisoning
Dinoflagellates: *Dinophysis *spp., *Prorocentrum lima*	Okadaic acids	Diarrheic shellfish poisoning
Dinoflagellate: *Protoperidinium *spp.	Azaspiracids	Azaspiracid shellfish poisoning
Dinoflagellates: *Gambierdiscus toxicus*, possibly *Ostreopsis *spp.;*Coolia *spp.; or *Prorocentrum *spp.	Ciguatoxins	Ciguatera fish poisoning
Cyanobacteria: *Microcystis*	Microcystins	Liver damage

HAB-related illnesses may be increasing in frequency [2]. This is in part because outreach efforts to the health care community have been successful, and health care providers are beginning to recognize and report HAB-related outbreaks to state health agencies. However, as HABs themselves appear to be increasing in frequency and duration, and as the number of people living in coastal regions grows [28], it is likely that more people will be at risk from exposure to HABs and their toxins, and thus at increased risk for developing HAB-related diseases.

Pathogens have been associated with a range of infectious diseases, manifesting as gastroenteritis, dermatitis, otitis, and upper respiratory illnesses (see Microbe Case Study). Anthropogenic chemicals, particularly POPs, have been associated with possible increased risks for immune and reproductive disorders as well as cancer in humans and marine mammals [19]. To date, significant health impacts from pharmaceutically active products (such as ingredients of birth control pills and of anti-depressant and anti-inflammatory medications) have been demonstrated only in non-human species including marine mammals; nevertheless, they may lead to as yet unidentified chronic health effects in humans as well [29].

Humans are exposed to water contaminated with chemicals and pathogens through skin and respiratory contact, and by eating contaminated seafood or other marine products. Thus, people are directly at risk from exposure to various contaminants bioaccumulated through the food web. In addition, humans are subject to indirect risks from these pollutants as a result of degraded marine resources, such as fish stocks [2]. Table 2 contains a brief summary of some waterborne pathogens and chemicals that pose risks to human health, an example of the source of the contaminant, and the symptoms or diseases they cause.

**Table 2:** Examples of microbial and chemical contaminants found in oceans that pose health risks to humans, possible sources of the contaminants, and typical symptoms or the disease induced by exposure to the contaminant.

**Contaminant**	**Possible source**	**Symptoms or disease**
Vibrio cholera	Human sanitary waste	Cholera
Norwalk Virus	Human sanitary waste	Gastroenteritis
Mercury	Burning coal, industrial use	Neurodevelopmental toxicity, adult toxicity
Persistent organic pollutants	Industrial waste	Immunologic, cancer, reproductive

One limitation in our ability to predict and prevent human health impacts from contaminated seafood/water is the lack of sensitive and specific biomarkers for contaminants. Several ongoing research activities seek to develop empirical biosensors to detect HAB species at low concentrations, to detect contaminants in seafood, and to evaluate human exposures. Such rapid detection technologies could be incorporated into "real-time" monitoring devices to be used in the field and in the clinic in the future [30].

### Socio-economic consequences

Research into the economic consequences of HABs, chemicals and pathogens on human health is in its infancy, but is particularly important in helping us to quantify the impacts of these ocean-associated agents. For example, Hoagland *et al. *[31] compiled estimates of the economic effects, including public health effects, of HABs for events in the U.S. where such effects were measured during 1987–1992. Total economic effects from HABs are estimated to be on the order of $50 million each year, of which public health effects account for about $20 million. While specific HAB events can have serious and significant economic effects at local levels, estimates of the scale of these effects must still be regarded as uncertain [32]. Given *et al. *[33] estimated that there were an excess 600,000 to 1,500,000 excess cases of gastroenteritis associated with microbial pollution at Southern California beaches per year, with an associated medical cost of $21–49 million.

Additional research needs to be done to estimate the economic impact of seafood contaminated with HAB toxins, pathogens and/or anthropogenic chemicals in terms of occupational and recreational losses as well as medical costs. Some of this work, for example on the social and economic consequences of ciguatera toxin exposure through seafood exposure and of aerosolized brevetoxin exposures, is now in progress. In some cases, there are broader social impacts beyond compromised health in an affected population associated with the loss of ocean resources. For example, the contamination of a seafood resource can lead to the loss of access to that resource for groups that have historically depended on it for nutrition and as part of traditional cultural practice [6,19-22].

The following paragraphs describe case studies focusing on two recently studied "new" issues in the linkage between oceans and human health: aerosolized brevetoxins and bacteria shed by bathers.

### HAB case study: aerosolized red tide toxins (brevetoxins) and asthma

As the incidence of asthma increases, there is increasing concern about environmental exposures that may trigger asthma exacerbations. Blooms of the marine micro-algae *Karenia brevis *cause Florida red tides, a type of harmful algal bloom (HAB), annually throughout the Gulf of Mexico. *K. brevis *produces highly potent natural polyether toxins known as brevetoxins. In animal experiments, brevetoxins have been shown to cause significant bronchoconstriction. In human epidemiologic studies, a significant increase in self-reported respiratory symptoms in humans has been described after recreational and occupational exposures to Florida red tide aerosols, particularly among asthmatics [34].

Before and after 1 hour on beaches with and without an active *K. brevis *red tide, 97 persons 12 years and older with physician-diagnosed asthma were evaluated by questionnaire and spirometry testing. Concomitant environmental monitoring, water and air sampling, and personal monitoring for brevetoxins were performed. Participants were significantly more likely to report respiratory symptoms after *K. brevis *red tide aerosol exposure than before exposure. Participants demonstrated small, but statistically significant decreases in their pulmonary function after exposure, particularly among those regularly using asthma medications. No significant differences in lung function were detected between pre- and post-beach-exposure periods when there was no Florida red tide.

This study demonstrated objectively measurable adverse changes in lung function from exposure to aerosolized Florida red tide toxins in asthmatics, particularly among asthmatics requiring regular asthma medications. Future studies will assess these susceptible subpopulations in more depth, examine possible sub chronic and chronic effects of these toxins, and help determine the clinical significance of these results.

### Microbe case study: quantitative evaluation of bacteria released by bathers in marine water

For many years, microbial contamination of recreational waters has been a source of public health concern. Enterococci bacteria have been used as a common fecal indicator; and *Staphylococcus aureus *bacteria are common skin pathogens with increasing antibiotic resistance. Both of these bacteria can be shed by bathers. It is assumed that when people shed enterococci, they may also shed other pathogens with which they are infected. Thus, the shedding of enterococci and *S. aureus *bacteria by bathers into recreational waters has negative connotations for human health. Recent studies have focused on estimating the amounts of enterococci and *S. aureus *shed by bathers directly off their skin. These studies were conducted at a marine beach located in Miami-Dade County, Florida [35].

Results from the first study demonstrated that bathers shed concentrations of enterococci and *S. aureus *on the order of 3 × 10^5 ^and 3 × 10^6 ^colony forming units (CFU) per person in the first 15 minute exposure period, respectively. Significant reductions in the bacteria shed per bather (50% reductions for *S. aureus *and 40% for enterococci) were observed in the subsequent bathing cycles. This suggests that bathers transport significant amounts of enterococci and *S. aureus *to the water column, and that human microbial bathing load should be considered as a non-point source when designing models of recreational water quality.

## Flooding

### Overview of public health consequences

In economic terms, floods – and their public health consequences – are jointly produced by humans and nature. Freshwater floods result from supra-normal rates of river and stream flows. Coastal flooding can be caused by surges that push marine waters onshore. Both freshwater overflows and coastal surges can be triggered by storm events (including hurricanes) that bring excessive rainfall or by other natural hazards (such as earthquakes), leading to dam failures upstream or tsunamis in the ocean. Humans co-produce the public health consequences of such hazards by living or working in harm's way [36].

Ahern *et al*. [37] find that floods are the most common natural hazard worldwide. Flood impacts can be mitigated by infrastructure (e.g., levees, dams) or institutional measures (e.g., building restrictions, insurance). Kahn [38] points out that fewer mortalities occur in developed nations, especially in democracies or nations with relevant institutional response capacity, during flood events.

On average, floods cost the United States about $6 billion each year and kill about 140 people [39]. Drownings are the single most common source of mortality (90%, by some estimates) from floods [40]. Worldwide, Jonkman and Kelman [41] observe that about two-thirds of flood-related fatalities involve drownings; and men, who tend toward risk-taking in such situations, are more likely to drown than women. These authors note also that a significant number of fatalities result from other causes, including dehydration, starvation, infections, injuries, and disease.

### Public health hazards from flooding

Marie [42] identifies a wide range of health hazards associated with the flooding caused by hurricanes. Physical hazards include drowning; contaminants in air, water, and soil; waterborne illnesses; fallen power lines; infestations of insects and other pests; and mold growth. Contaminants comprise raw sewage (which affects drinking water and food), as well as toxins (such as lead dissolved from housepaint or arsenic leached from soils).

Waterborne illnesses may be caused by a wide range of bacterial and viral pathogens, and include cholera, salmonellosis, amebiasis, campypylobacteriosis, cryptosporidosis, hepatitis A, shigellosis, and viral gastroenteritis. Mold growth on wet surfaces can produce infections and allergies, and release mycotoxins. Standing water can be a breeding ground for pests, especially mosquitoes, leading to the spread of West Nile virus, encephalitis, malaria, or other vector-borne diseases.

Human hazards include human responses to impaired public services and psychological impacts [42,43]. The lack of potable water can lead to waterborne illnesses, if tainted water is consumed, or dehydration. The inability to access public services (such as electricity or fuel) may lead to the use of fuel-burning devices in poorly ventilated areas, causing carbon monoxide poisoning. Communicable diseases can be spread more rapidly when humans are billeted in the close quarters of emergency shelters [42,43].

Emotional or psychological impacts can result from the loss of homes, properties, or the deaths or sicknesses of friends and family. Psychological impacts are manifested in depression, anxiety, grieving, shock, insomnia, moodiness, substance abuse, or marital problems. The Harvard Medical School's Hurricane Katrina Community Advisory Group found that, as post-traumatic stress reactions, mental illnesses were twice as prevalent in surveyed survivors as they were in the pre-hurricane New Orleans population [44].

### Need for epidemiological studies

While the list of potential public health effects is extensive, careful studies of the frequency and incidence of such effects are rare. For example, Ahern *et al. *[37] review the literature on the epidemiological evidence for the health effects of floods, looking at cases from all over the world. The authors found that flood impacts depend critically upon the type of flood and the vulnerability of the affected population. Floods leading to the largest numbers of deaths tend to be those that either inundate a population with limited economic resources or for which the infrastructure for responding to the hazard is inadequate. However, there is limited evidence on the health effects of floods, especially the effects that point to illnesses as opposed to deaths [37]; and research to date is insufficient to establish links between flood-induced chemical contaminations and either morbidities or mortalities [43].

There is a critical need for epidemiological research to establish the public health consequences of flooding in coastal and inland environments. Ahern *et al. *[37] identify epidemiological knowledge gaps concerning the causes and long-term effects of mental health impacts, the nature and magnitude of mortality risks, the risks of infections and vector-borne diseases, the effectiveness of warning systems and public health measures, and the extent to which flood risks and health burdens are affected by climate and land-use changes.

Better epidemiological studies on the health effects of flooding will require data on flood losses. In the United States, there is no single government agency with responsibility for compiling such data [45]. At least three national databases include potentially useful data on the economic effects of floods. These databases are the National Weather Service's (NWS) "national flood damages" (1926–2007) [45]; the National Hurricane Center's (NHC, a bureau of NWS) "deadliest, costliest, and most intense US tropical cyclones" (1856–2006) [46]; and the Federal Emergency Management Agency's (FEMA) "significant flood events" (1978–2007) [47].

**Figure 2:**
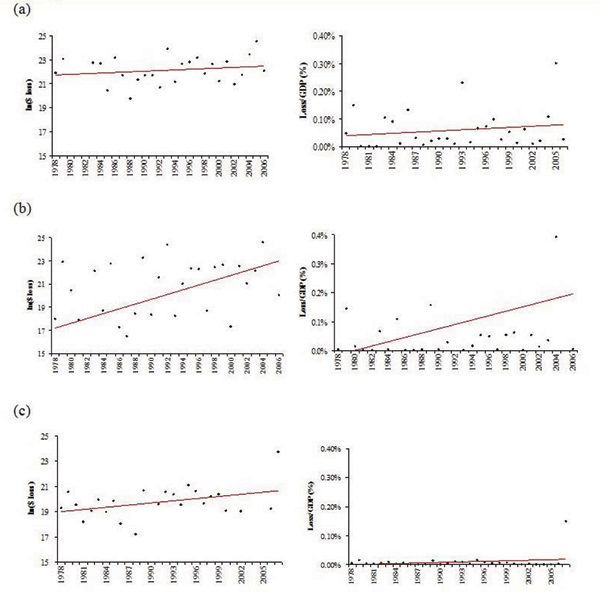
**Estimates of economic losses from natural hazards involving floods from three national databases**. Figures on the left represent the natural logarithm of annual losses; figures on the right represent these losses as a percent of the US gross domestic product. All estimates have been converted to 2007 dollars using the consumer price index. Data are compiled from: (a) national flood damages (excluding those associated with coastal storm surges); (b) losses from the deadliest, costliest, and most intense US tropical cyclones; and (c) paid flood insurance losses from significant flood events. Please see the text for a description of coverage, gaps, and overlaps.

Most analysts agree that there is significant error in the estimates of national flood losses [48,49]. However, there is no evidence of systematic bias in estimated losses [50]; and these databases are regarded as roughly indicative of *trends *in economic losses over time, if not accurate representations of the actual amount of losses in any particular year or for any particular event. Figure 2 displays annual estimates of flood losses from each of the databases and relates flood losses to the growth of the national economy, as measured by gross domestic product. Although flood losses continue to grow over time, and may be significantly influenced by extreme events such as Hurricane Katrina, national flood damage losses as a proportion of national GDP (Figure 2(a)) appear to increasing only slowly.

More problematic for understanding the human health implications of coastal flooding is that these databases mingle estimates of different kinds of losses. All three are focused on direct impacts (i.e., property losses) as reflected in insurance payments or the cost of the repair of public infrastructure; they usually do not include indirect (secondary or tertiary) impacts, which include morbidity and mortality estimates, among other potential losses [51]. And there has been little if any research on the environmental benefits and costs of flooding [48] (see Katrina Case Study).

As an example, many experts estimate the range of economic losses from Hurricane Katrina to be between $100 to $200 billion [45,39]. Taking $125 billion as an average estimate, NWS's Hydrologic Information Center estimates that 60 percent (~$75 billion) of these losses were attributable to the storm surge, 30 percent (~$37.5 billion) to flooding; and ten percent (~$12.5 billion) to wind damage. Thus, only the $37.5 billion would appear in the national flood damage database. An estimate of $16 billion appears for Katrina as paid losses in the FEMA significant flood database. NHC's estimate of the cost of Katrina as a tropical cyclone is $81 billion, differing significantly from the other two agency estimates and much lower than the expert estimates.

### Flooding case study: Katrina

Great concern was expressed regarding the potential public health impact of communicable diseases (such as cholera) after Hurricane Katrina made landfall in September of 2005. Fortunately, there were no large scale outbreaks of communicable disease, but there has been increasing recognition of effects on the environmental health of New Orleans, particularly in low-lying areas that flooded by adjacent Lake Ponchartrain. In a collaborative study investigating Lake Pontchartrain's microbial environment over a period of about 1.5 years following the city's flooding, research showed that this environment returned to pre-storm (but not clean) conditions within about 2 months after floodwaters from the city were pumped back into Lake Pontchartrain.

In addition to testing water samples from Lake Pontchartrain and the canals draining from the city into the lake, this work also examined sediments deposited by the hurricane floodwaters around homes and other sites within the city. The persistence of high levels of indicator organisms in these sediments and soils up to 8 months after the flooding suggests that there may be additional public health issues from exposure to these contaminated soils. Molecular source-tracking indicated that at least a portion of the indicator organisms detected in near-shore lake waters, canals, and sediments came from human fecal origins, suggesting that the microbial contamination seen in the floodwaters and deposited sediments resulted from the interaction of the floodwaters with the challenged sanitary infrastructure of the city itself, a sanitary infrastructure which continues to negatively impact the near-shore environment of the city long after the floodwaters have receded [52].

## Nutritional benefits of seafood consumption

Although seafood may accumulate contaminants from natural toxins and toxicants, there are numerous health benefits from seafood consumption. Seafood, both finfish and shellfish, is an important source of protein, essential fatty acids, and micronutrients (such as Vitamin D, iron, zinc, selenium, and iodine). In the U.S., approximately 4% of total protein intake currently comes from fish and shellfish [53]. Compared to other foods that are high in protein (such as meats, poultry, and dairy), seafood contains relatively low levels of calories and saturated fat. In summary, seafood is an important source of essential nutrients for humans, and for many coastal and island populations, the major source of protein (see Seafood Case Study).

Seafood is also an important source of essential polyunsaturated fatty acids (PUFAs), specifically omega-3 PUFAs. In particular, docosahexaenoic acid (DHA) and eicosapentaenoic acid (EPA) are types of omega-3 PUFAs available to humans through seafood consumption [54]. The myriad health benefits of DHA and EPA have been studied in humans and other animals; these include benefits for the cardiovascular system, nervous system, development, and immune system [55-60]. Because of the strong beneficial effects from consuming omega 3 PUFAs, the American Heart Association recommends eating at least two servings of fish per week to prevent cardiovascular diseases [61]. EPA and DHA supplementation is also recommended for patients with elevated triglycerides and/or coronary heart disease [61].

Maternal consumption of fish and/or fish oil has been associated with improved neurodevelopment and birth outcomes [57,60]. Other positive effects of DHA supplementation during the perinatal period on the mental development of the fetus and newborn have been shown. For example, Helland *et al. *[62] found that children whose pregnant and lactating mothers were randomized to eat fish oil scored higher on an IQ test at age 4. Most studies investigating connections between omega-3 PUFA intake and birth outcomes (such as gestational age, fetal growth, and infant size) suggest that higher intake of omega-3 fatty acids may indeed improve birth outcomes [62,63].

The consumption of seafood varies depending on culture, geography, and economic status in the U.S. Fish and shellfish have enormous cultural importance among Pacific Northwest Tribal Nations, Asian-Pacific Islanders, and other U.S. population groups [19]. Previous studies among these groups have found consumption levels up to ten times larger than the average U.S. consumer [19-22]. In addition to high consumption levels, it is important to consider cultural and lifestyle factors when assessing seafood consumption among different groups [19,64]. For example, organs such as the crab hepatopancreas concentrate certain toxicants, such as polychlorinated biphenyls (PCBs) [65], and are commonly eaten together with the rest of the crab and the cooking water by the Asian-Pacific Islander community [22], resulting in higher exposures to PCBs [4].

Knowing the source of the seafood is essential in assessing exposure to toxins or toxicants. In the Pacific Northwest, tribal groups typically gather their own seafood from local rivers, Puget Sound, or the Pacific Ocean [19], while Asian-Pacific Island groups primarily eat commercially caught fish from all over the world [22]. Fish sold commercially is subject to monitoring by the U.S. Food and Drug Administration; but FDA samples only a portion of the fish sold commercially to ensure that it meets standards. Locally caught fish varies greatly in contaminant levels as different lakes, rivers, and coastal areas have unique levels of PCB contamination [19].

### Seafood case study: assessing exposure from diet

Seafood consumption rates vary greatly across demographic groups, with some groups consuming up to ten-fold higher levels of seafood than the average U.S. population [19-22,66]. A challenge in determining potential risks from eating contaminated seafood is the uncertainty involved in exposure assessment. Several tools are available to assess exposure [67]. The "gold standard" is the "diet diary," where trained individuals record participants' intake at the time of consumption. This method involves significant costs and requires both exceedingly motivated subjects and well trained researchers since variability increases if dietary data are not recorded or interpreted in a consistent manner. Dietary recalls are commonly used in clinical settings and by researchers to assess dietary intake; a trained researcher/dietician asks what the individual has eaten in the last 24, 36 or 72-hour period. However, this method captures only a snapshot of the diet and may not be representative of the individual's usual intake. A preferred tool to capture long-term consumption patterns is the food frequency questionnaire (FFQ), which obtains information on frequency and portion sizes of food items of interest over a defined period of time [68].

## Implications for management and research priorities

Ultimately, we must strive for clean coastal and marine waters with a safe food supply, all of which support the health of both humans and other animals (including susceptible populations). The effective and efficient application of resources to managing human and public health risks associated with the oceans requires an understanding of the physical, biological, chemical, behavioural, and economic dimensions of the interactions of humans with ocean-related health risks.

The research directions outlined here build on other recent work on this topic, including a framework for research and monitoring articulated by an international group of researchers [69], and a set of priorities formalized in the "Oristano Declaration" at an international workshop on "Marine-based Public Health Risk" in Sardinia in 2003 [70,2]. These earlier efforts took a global perspective on risks primarily from seafood and from direct exposure to marine water, and emphasized the importance of international cooperation on surveillance and risk assessment for changes in the marine environment and for human health effects. They also called for research to focus on techniques for early detection and rapid assessment of marine environmental contaminants and risks.

Our assessment of the implications for management of and research on human health effects from marine sources of risk is consistent with these prior efforts. We take a slightly broader perspective on the spectrum of risks (e.g., explicitly including flooding events), and place greater emphasis on the social science work required to properly anticipate the human response to these risks, and to design appropriate management and mitigation measures.

### HABs, pathogens, and other pollutants

From the point of view of management, any human activity that adversely affects the quality of ocean waters or coastlines, or that increases human exposure to chemicals or pathogens, should be evaluated and, if necessary, modified to prevent and mitigate exposure risks.

The extent to which human activities have led to an apparent increase in the number, intensity, and duration of HABs is currently a source of considerable debate; however, for certain species (e.g., the cyanobacteria) there is a clear connection between nutrient loading and subsequent blooms that may be accompanied by toxin production. Therefore, the regulation of nutrient contamination (particularly from non-point sources) of marine and coastal waters may be an important intervention to reduce the impact of HABs. For both the pathogens and the anthropogenic chemicals, preventing dumping of waste into the oceans is one obvious way to mitigate human exposure to ocean-borne toxicants. Recent evidence indicates that non-point sources (such as urban and agricultural run-off) may also be significant sources of ocean-borne pollutants, including chemicals and pathogens. Removing these sources of contaminants is often more difficult or expensive than preventing point-source pollution, but would certainly contribute to mitigating public health risks from ocean-born contaminants. More research is needed to quantify the impact of non-point source pollution on the health of both the oceans and of humans, as well as practical methods to address it.

### Seafood

A central issue in managing risks associated with seafood consumption concerns risk perception, risk response, and tradeoffs between positive and negative effects. For example, there is an inherent conflict between public health messages warning certain subpopulations about the risks of eating contaminated seafood (due to the real and apparent dangers associated with contamination by HAB toxins, microbes or anthropogenic chemicals), and public health messages that encourage people to eat fish as a source of high-quality protein and other nutrients, such as omega-3 fatty acids [71,72].

### Flooding

To date, the majority of the research on the public health effects of flood events has addressed freshwater flooding in river basin environments. Relatively little effort has been devoted to the systematic study of public health effects of coastal flooding with marine or estuarine waters. The relative lack of attention to coastal flood hazards may due in part to the limited effect of the most common coastal flooding events and the rare, episodic nature of the large-scale catastrophic events, such as the inundation that occurred in southern Louisiana with Hurricane Katrina.

The broad categories of research needs relating to the public health consequences of coastal flooding include: (i) epidemiological studies of the risks of morbidities and mortalities; (ii) the scales and time trends of economic losses; (iii) the costs and effectiveness of management measures; and (iv) integration of scientific, economic, and epidemiological research with decision-making. To a significant extent, research in these categories will depend upon both the nature of the storm and the special characteristics of each location, including the vulnerability of the population at risk, the physical characteristics of the location, and the existing structural and institutional management measures that are in place. Of particular concern is the development of reliable estimates of the economic losses from coastal flooding, which are not now compiled routinely and consistently. Understanding so-called secondary effects, comprising public health costs among other types of costs, is an urgent need. With reliable data on costs, private and public planners and decision makers can begin to evaluate the benefits and assess the appropriateness of alternative management responses.

### Acute, subchronic, and chronic health effects

We know little about the subchronic and chronic health effects resulting from exposure to water-borne HAB toxins, pathogens, or anthropogenic chemicals. The economic and societal impacts associated with acute and chronic (particularly low dose and mixed) exposures to all these threats have not been completely characterized, and without this quantification of impact, it is difficult to bring attention and resources to these potentially important health risks.

### Prevention and mitigation

Finally, solutions to prevent and mitigate the known effects of the HAB toxins, pathogens and anthropogenic chemicals are needed. The needed solutions will range from recreational beach warnings and forecasts, to the assessment of land use practices to enhance protection of watersheds and coastlines, to medications that specifically block the effects of the HAB toxins or other biologically active ocean-borne contaminants. The design of management measures must take into account the complexities of human response to warnings and other guidance, and the economic tradeoffs among different risks and benefits (e.g., seafood consumption or beach recreation).

Researchers including Morss *et al*. [73] have emphasized a critical need to integrate the conduct of scientific research with decision-making. All too often, scientists view public decision makers as one coherent entity. In reality, those officials tasked with responding to ocean health hazards ("practitioners") have many different responsibilities to be undertaken at many different levels of government. This administrative fragmentation implies that practitioners will have varying knowledge and information requirements. Furthermore, whereas scientists may be concerned with the compilation and analysis of data and the testing of hypotheses to reduce uncertainty about effects, practitioners are also concerned about community perceptions of risk and the political acceptability of alternative management measures. Ignorance about the realities of public administration implies that scientific research often may lack relevance and its results may not be fully utilized. Morss *et al*. [73] suggest that scientific research needs to be coupled more closely to the needs of the practitioner on a continuous basis. Following this approach, scientists and practitioners would collaborate on the design and implementation of research agendas with the goal of improving decision-making about managing public health risks.

### Ecosystem perspective

Several of the ocean-public health links described in this paper involve adverse health effects caused by environmental conditions, events, or agents directly causing physical harm or disease. Understanding the etiology of injury or illness in an environmental health context requires investigating social and ecological as well as biological origins, pathways, and mechanisms leading to illness or injury. The need for an ecosystem-based approach is amplified by the accelerated changes taking place in the oceans as a result of global change, and the implications of this for environmental health management.

Large-scale environmental change may play an increasingly important role in ocean-public health links, and is thus a significant emerging concern. With the exception of anthropogenic toxins, the health hazards we have discussed are primarily of natural origin. Yet all are (potentially) amplified by large-scale regional or global environmental change, involving a complex mix of social and environmental modifications resulting from human activities largely associated with globalization [74]. These include: global warming, depletion of the ozone layer, resource depletion (renewable and non-renewable), loss of biodiversity, urbanization, and widespread environmental pollution.

In the marine and coastal context, evidence increasingly suggests that these changes may exacerbate ocean-public health hazards. Examples include the negative effects of coastal development on the capacity of coastal catchments, wetlands, and estuaries to modulate and mitigate the effects of nutrient, sediment, and toxic chemical-bearing runoff; the effect of the increasing frequency or severity (or both) of storm events on the health consequences related to flooding and storm surges; and changes in the range of marine organisms associated with toxins or pathogens as a result of climate change or anthropogenic nutrient loading of coastal waters.

The effects of ecological degradation on a regional scale have been documented in terms of losses in native species diversity and reduced ecosystem processes and services [75]. General examples include reductions in water filtering and detoxification by suspension feeders, submerged vegetation, and wetlands; and the increasing occurrence of harmful algal blooms, fish kills, shellfish bed and beach closures, and oxygen depletion. Increasing coastal flooding events may be linked to sea level rise, but also probably accelerated by historical losses of floodplains and erosion control provided by coastal wetlands, reefs, and submerged vegetation.

These findings suggest that in addition to conventional environmental health and environmental quality management approaches (such as risk communication and controlling the environmental release of pollutants), approaches that consider the maintenance of ecosystem processes are critical. In particular, there is a growing consensus, originally suggested as one of the basic elements of ecosystem management [76], that policy and management actions should center on protecting ecosystem "resilience" [77]. Resilience is described as an inherent property of intact ecosystems that represents the system's capacity to assimilate disturbances or stresses, and recover [78]. More recently, disease ecologists have begun to associate the resilience present in an intact ecosystem, and conversely its loss, with the regulation of pathogen emergence [79] and with implications for humans [80,81]. There is also growing interest in fostering the resilience of human coastal communities to short-term hazards and long-term changes [82].

Thus, the maintenance of resilient ecosystems and their capacity to ameliorate harmful natural and anthropogenic environmental conditions and events, and regulate pathogens, is a critical component of an ocean-public health risk management strategy.

## Conclusion

The systematic study of linkages between the oceans and human (public) health comprises a new, interdisciplinary field. Exposure to coastal waters and interactions with marine resources can have both positive and negative human health effects. Major linkages include exposure to HABs and their toxins, microbes, and chemical pollutants; recreational or occupational contact with ocean waters; consumption of seafood; coastal flooding; and other exposure pathways. Health effects in a given population are determined by a complex interaction of exposure and susceptibility.

The state of present knowledge about the linkages between oceans and public health varies. Some risks, such as those posed by HAB toxins associated with shellfish poisoning and red tide, or consumption of seafood contaminated with heavy metals, are relatively well understood. Other risks, such as those posed by chronic exposure to many anthropogenic chemicals, pathogens, and naturally occurring toxins in coastal waters, are less well quantified. Even where there is a good understanding of the mechanism for health effects (e.g. with ciguatera toxin or exposure to pathogens associated with sewage), good epidemiological data are often lacking. Solid data on economic and social consequences of these linkages are also lacking in most cases. New collaborative research initiatives involving oceanographers, biologists, social scientists, and epidemiologists are beginning to address these data gaps and are well positioned to facilitate the integration of epidemiological and socio-economic work with the biology and chemistry of human exposure to marine pathogens and toxins.

Future public health research priorities should include epidemiological studies in collaboration with public health agencies to better understand human health effects at the population scale, as well as systematic economic work to support, in conjunction with the biological and chemical science, effective and efficient management measures. Finally, because the study of linkages between public health and the oceans is a new field, emphasis must also be given to education and training of future researchers in oceans and human health.

## Competing interests

The authors declare that they have no competing interests.
